# A Pandemic Paradox: Innovations in Psychological Resiliency and Suicide Prevention in Older Adults

**DOI:** 10.1093/geroni/igab046.1608

**Published:** 2021-12-17

**Authors:** Marnin Heisel

**Affiliations:** Schulich School of Medicine & Dentistry, The University of Western Ontario, London, Ontario, Canada

## Abstract

The COVID-19 pandemic has had a substantial negative impact on the health and well-being of older adults, a demographic with the highest proportion of fatalities in North America. Long-term care and retirement homes have been especially hard hit. Sheltering in place can increase social isolation among older adults and contribute to feelings of stigmatization, burden, stress, anxiety, anger, and despair. As older adults also account for high rates of suicide, fear of infection, reduced access to professional and social supports, and growing apathy, hopelessness, and social isolation could amplify suicide risk (see Zortea et al., 2020). The speaker will discuss how his program of research on psychological resiliency and suicide prevention in older adults has pivoted online over the past year, and how the pandemic has paradoxically inspired innovative approaches to research, education, clinical practice, and social advocacy for older adults that will likely continue well beyond the present time.

